# Data on social and health vulnerability in rural India: A case of covid-19

**DOI:** 10.1016/j.dib.2020.106020

**Published:** 2020-07-14

**Authors:** Surendra Singh

**Affiliations:** ICAR- National Institute of Agricultural Economics and Policy Research, Agricultural Economics, DPS Marg PUSA, New Delhi, 110012, India

**Keywords:** Covid-19, Livelihood vulnerability, Rural economy, Health vulnerability, Regional inequalities

## Abstract

An attempt was made to understand the gap between health and social vulnerability to Covid-19 pandemic. By using multistage sampling technique, 150 samples were collected during March- April 2020. Data highlight that households are highly exposed to novel Covid-19, and also equally sensitive to inadequate and poor availability and accessibility of clean water, sanitation and health care system. Data suggest a grass root awareness program (*ex-ante* preventive measures) in the regional languages; increase public health budget to meet the demand and improve the supply chain; establishment of more physical and human infrastructure in long run; comprehensive plan to ensure continuous water supply in the rural areas.

Specifications table

**Table:** 

Subject	Social Science (Economics)
Specific subject area	Household's livelihood vulnerability to Covid-19
Type of data	Table Figure
How data were acquired	• After in-depth discussion with doctors, academicians, a structured schedule was develop. Questions were framed in such a way to capture the household's perception to Covid-19, such as do you perceived that frequency of health-hazards increased over past five years. • Face-to-face interview was conducted. • Excel 2013 software was used for data coding, cleaning and analysis.
Data format	Data are in raw format (Excel 2013). An excel file with raw data has been uploaded
Parameters for data collection	Household's responses such as do you perceived that frequency of health-hazards increased over past five years was converted into percentage form at village-level. After that, percentage values were converted into index scores using [1] methodology.
Description of data collection	• After taking expert advice from doctors, academicians and public administration, a structured scheduled was prepared to elicit household's perception to Covid-19, their socioeconomic status and curative and preventive measures they have taken. • Field survey was undertaken during March- April 2020. Survey was conducted during nationwide lockdown. Therefore, special permission for field survey data was taken to local administration to elicit the data. Head of village (i.e., secretory) was contacted for the sample survey. • A multistage sampling technique was opted to collect household-level data. In first step, most populous state out of 36 states and union territories was selected, i.e., Uttar Pradesh. In the second step, 1 district out of 72 district was randomly selected, i.e. Mathura. In the third step, 5 out of 254 villages were randomly selected, i.e., Virjapur, Nawada, Narholi, Adooki and Bad. In the fourth step, 30 samples from each village were collected using random sampling technique. Thus total 150 samples were collected in five villages of Mathura district to elicit the households’ perception of Covid-19 regarding preventive and curative measures used by the villagers to cope with Covid-19.
Data source location	City/Town/Region: Mathura, Uttar Pradesh Country: India Latitude and longitude for collected samples/data: 27.49° N, 77.67° E
Data accessibility	Raw data has submitted

**Values of Data**

• These data are unique and relevant for health policy for rural India because they provide village-level vulnerability status of households to health emergency, i.e., Covid-19 pandemic.
• These data are useful for social scientist and policy makers because they highlight that how a rural-household responded against health emergency and how a household optimize their resources to cope with it.
• By using these data, policymaker can develop a rapid health contingency response plan to deal with any future health emergency, like Covid-19 pandemic.
• The additional value of these data are that they cover socioeconomic dimension of households, their coping behavior and reliance on the public health system against to Covid-19 pandemic.

## 1 Data description

After taking expert advice from doctors, academicians and public administration, a structured scheduled was prepared to elicit household's perception to Covid-19, their socioeconomic status and curative and preventive measures they have taken. Field survey was undertaken during March-April 2020. Survey was conducted during nationwide lockdown. Therefore, special permission for field survey data was taken to local administration to elicit the data. Head of village (i.e., secretory) was contacted for the sample survey. Face-to-face interview was conducted. A multistage sampling technique was adopted to collect household-level data. In first step, most populous state out of 36 states and union territories was selected, i.e., Uttar Pradesh. In second step, 1 district out of 72 district was randomly selected, i.e. Mathura. In third step, 5 out of 254 villages were randomly selected, i.e., Virjapur, Nawada, Narholi, Adooki and Bad. In fourth step, 30 samples from each village were collected using random sampling technique. Thus total 150 samples were collected in five villages of Mathura district to elicit the households’ perception of Covid-19 regarding preventive and curative measures used by the villagers to cope with Covid-19.

## 2 Experimental design, materials and methods

The main aim of these data was to integrate both health and social vulnerability indicators in Livelihood Vulnerability Index (LVI) applicable at any scale and capable of identifying household's vulnerability and the most vulnerable members of society. Face-to-face interview was conducted to collect household's responses to Covid-19. After in-depth discussion with doctors and mainstream researchers; questions were categorically asked to the respondents that do you perceived the frequencies of health-hazards have increased over past five years. Thereafter, data was codded into numerical values, such as 1 for household has perceived that frequencies of health-hazards have increased over past five years, while 2 for household don't perceived that frequencies of health-hazards have increased over past five years. Village average was used to develop exposure, sensitivity and adaptive capacity indices for different villages. Further, Hahn et al. [1] methodology was used to the normalized values, while Singh [2] methodology was used to develop a livelihood vulnerability index for different villages using Excel 2013 software.

**Fig. 1:**
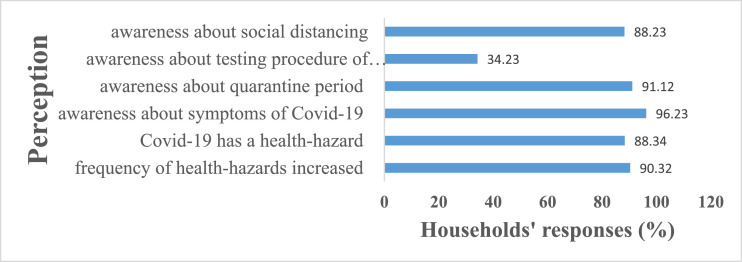
Households’ perception of Covid-19

Source: Field survey, 2020.

Data show that sample households are well-aware of Covid-19 pandemic. About 91.12% of households are well-aware of quarantine period, and about 96.23% of households are well-aware about the symptoms of Covid-19 (Fig. 1). Also, household have knowledge of social distancing (i.e., 88.23%), while households marginally has awareness about testing procedure of Covid-19.

**Fig. 2:**
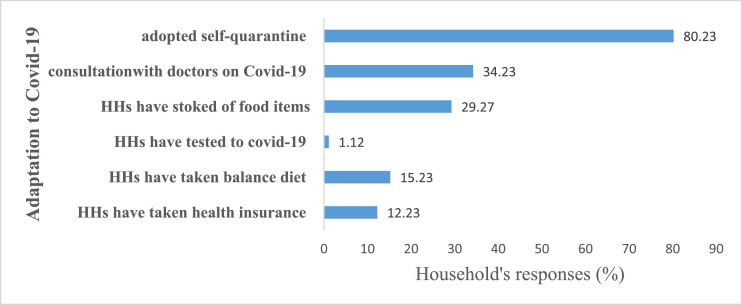
Adaptation strategies to Covid-19

Source: Field survey, 2020.

Additionally, about 80.23% of household went of self-quarantine, while about 34.23% of households have consulted to doctors over Covid-19 symptoms (Fig. 2). As government has imposed 21 days complete lockdown, except essential items, about 29.27% of households have stored food items to deal with health emergency. By adopting health advisory issued by World Health Organization (2020), about 15.23% of households have taken balance diet, while about 12.23% of households have taken health insurance which covers Covid-19. Moreover, about 1.12% of households have tested to Covid-19. In totality, in the absence of effective vaccine, preventing measures, such as self-quarantine was first choice to deal with Covid-19.

Data of exposure indices for households belonging to the Virjapur village indicate that they are highly exposed to Covid-19 than that of other villages (Table 1). Data of sensitivity indices for households belonging to the Virjapur village also relatively high, whereas these households had a lower adaptive capacity. In totality, households belonging to Virjapur village are relatively highly vulnerable than others.

**Table 1:** Village wise livelihood vulnerability index.

Sub-components	Virjapur	Nawada	Narholi	Adooki	Bad
Exposure Index	0.76	0.56	0.60	0.71	0.64
Sensitivity Index	0.41	0.37	0.33	0.30	0.23
Adaptive Capacity Index	0.22	0.31	0.37	0.42	0.45
Livelihood Vulnerability Index	0.221	0.092	0.076	0.088	0.043

## Declaration of Competing Interest

No potential conflict of interest was reported by the author

## Appendix Supplementary materials

Image, application 1

Image, application 2
