# Supplementary material for: Data on social and health vulnerability in rural India: A case of covid-19
Source: Data Brief. 2020 Jul 14;31:106020. doi: 10.1016/j.dib.2020.106020 (PMC7358164; doi:10.1016/j.dib.2020.106020)
Supplement: Supplementary file 2 [file mmc2.docx]

**Data on social and health vulnerability in rural India: A Case of Covid-19**

**1.** **Experimental design, materials and methods**

The main aim of these data was to integrate both health and social vulnerability indicators in Livelihood Vulnerability Index (LVI) applicable at any scale and capable of identifying household’s vulnerability and the most vulnerable members of society. The indicators were normalized so as to use a single scale based on their functional relationship with vulnerability: equation (1) was used for a positive relationship with vulnerability and equation (2) was used for a negative relationship with vulnerability (Hahn et al., 2009):

${Index}_{sv}=\frac{S_{v}-S_{min}}{S_{max}-S_{min}}$……………….(1)

${Index}_{sv}=\frac{S_{max}-S_{v}}{S_{max}-S_{min}}$……………….(2)

Where, $S_{v}$ is the raw value of the indicator at household level; and $S_{min}$ and $S_{max}$ are the minimum and maximum values of the indicator across all households (Singh, 2020). In this way the indicators were normalized on a scale of 0 to 1.

The present study uses the normalized values of households’ perception of Covid-19 as proxy indicators to calculate an exposure index, normalized socioeconomic indicators to develop a sensitive index, and normalized adaptation strategy indicators to a develop an adaptive capacity index (Table 1) using equation (3-5) as follows:

${Exposure}_{index}=\frac{FHZ+HZ+Sym+Qua+Test}{5}$…… (3)

${Sensitivity}_{index}=\frac{BPL+Migration+Returned+ASH+ACW+AS+Illitrate+FHH+Ele+GHS+Social+FAG+Food+PGL+FBA}{15}..$(4)

${Adaptive Capacity}_{index}=\frac{Income+Land+Insurance+Diet+TAC+ARD+Stock+Ypop+Doctors+ASQ}{10}..$(5)

Where, the variables on the right-hand side are normalized version of the indicators listed in Table 1.

Once the value of exposure, sensitivity and adaptive capacity for the household level had been calculated, Singh (2020) methodology was opted to calculate household-level livelihood vulnerability index using equation 6 as follows:

$${LVI}_{d}={(Exposure}_{d}-{Adaptive Capacity}_{d})*{Sensitivity}_{d}$$

Where, ${LVI}_{d}$ is the livelihood vulnerability index for the d^th^ households, and ${Exposure}_{d}$, ${Sensitivity}_{d}$, &${Adaptive Capacity}_{d}$ are exposure index, sensitivity index and adaptive capacity index for the d^th^ household.

*2. Selection of indicators of exposure, sensitivity and adaptive capacity*

As mentioned in the introduction, this research sought to identify three dimensions of vulnerability, namely exposure, sensitivity and adaptive capacity of the respondents (Table 1).

Exposure refers to the impact of changes in the frequency, intensity/magnitude, duration and nature of health-hazard (IPCC, 2001). According to the District Health Department, Mathura district has a long-history of short to long- chronicle diseases. Malaria and viral fever are common infection diseases reported over the past few decades (GoI, 2019). Also, district is highly exposed to Covid- 19 due to semi-rural-urban settlements. Hence, perception of head of household on Covid-19 was taken as a proxy indicators. Questions such as: (i) what he/she has perceived of Covid-19, (ii) how he/she decides to present them, and (iii) does he/she perceived that Covid-19 has health-hazard?

Sensitivity is the degree to which the system is affected, either adversely or beneficially, by the health-hazard (IPCC, 2001) and the socioeconomic and ecological ability of a system to respond to hazard. The population below poverty line (BPL) is a section of the population that is deprived of resources and highly sensitive to hazard. In rural areas, Abid et al (2015) found that a higher dependency on the household head, low diversification in agriculture and a lack of non-farm employment opportunities and migration are key indicators influencing households to choose adaptation measures. Subsequently, Miranda et al (2011) found that vulnerable households spend 3-5 hours fetching drinking water from unsafe sources and also reported sharper conflicts over the use of water resources due to the water scarcity, depletion and poor access in the off-rainy season. Further, illiterate households were at greater risk, indicating that education increases the capacity to cope with water-induced. Also, the literature has shown that female-headed households are more vulnerable and less adaptive that male-headed households (Islam et al., 2013 and Nadeem et al., 2009).

Although the system might be significantly exposed or sensitive to health-hazard, it cannot be said that it is vulnerable (Fellmann, 2012). A system adaptive capacity influences vulnerability by adjusting both exposure and sensitivity (Gollopin, 2006). Successful and efficient adaptation is determined by three important factors: (i) timely perception and realization of changes in health-hazard and the need to adopt measures to adapt, (ii) incentives to adapt and ability to adapt (iii) need to alter the coping health practices. Masud et al (2017) suggested that adaptation to hazard is a prerequisite to reduce its negative impact and harvest the benefits of adaptation.

**Table 1: Selected rational indicators for livelihood vulnerability index**

| Sub-components | Indicators | Functional relationship |
| --- | --- | --- |
| Exposure | HHs perceived that frequency of health-hazards increased over past five years (%) (FHZ) | + |
|  | HHs perceived that Covid-19 has a health-hazard (%) (HZ) | + |
|  | HHs know about the symptoms of Covid-19 (%) (Sym) | + |
|  | HHs know about the quarantine period (%) (Qua) | + |
|  | HHs know about the testing procedure of Covid-19 (%) (Test) | + |
| Sensitivity | HHs belongs to below poverty line (%) (BPL) | + |
|  | Household head migrated in search of employment in the  Covid-19 affected cities (%) (Migration) | + |
|  | Household head returned from Covid-19 affected cities (%) (returned) | + |
|  | HHs don’t have all seasonal house (%) (ASH) | + |
|  | HHs don’t have access of clean water (%) (ACW) | + |
|  | HHs don’t have access of sanitation (%) (AS) | + |
|  | Household-head has illiterate (%) (illiterate) | + |
|  | Female-headed households (%) (FHH) | + |
|  | HHs don’t have electricity connection (%) (Ele) | + |
|  | HHs don’t have access of government health services (%) (GHS) | + |
|  | HHs belongs to backward social group (%) (Social) | + |
|  | HHs received financial assistance from government during lockdown (%) (FAG) | + |
|  | HHs get food during lockdown (%) (Food) | + |
|  | HHs participated in public gathering during lockdown (%) (PGL) | + |
|  | HHs don’t have functional bank account (%) (FBA) | + |
| Adaptive capacity | Household head has assured income (government employee) (%) (Income) | + |
|  | HHs have agricultural land (%) (Land) | + |
|  | HHs have taken health insurance (Insurance) | + |
|  | HHs have taken balance diet as a combat strategy (%) (Diet) | + |
|  | HHs have tested against covid-19 (%) (TAC) | + |
|  | Household head don’t affected to respiratory diseases (ARD) | + |
|  | HHs have stoked of all necessary food items before lockdown period (Stock) | + |
|  | HHs have majority (more than 80%) of young population (%) (YPop) | + |
|  | HHs have frequently consulted with doctors on Covid-19 (Doctors) | + |
|  | HHs have adopted self-quarantine during lockdown period (ASQ) | + |

Source: Author’s estimation, 2020, Note: HHs indicate households.

**Table 2: Village wise exposure index**

| Indicators | Virjapur | Nawada | Narholi | Adooki | Bad |
| --- | --- | --- | --- | --- | --- |
| HHs perceived that frequency of health-hazards increased over past five years | 0.83 | 0.67 | 0.68 | 0.79 | 0.88 |
| HHs perceived that Covid-19 has a health-hazard | 0.92 | 0.59 | 0.88 | 0.96 | 0.58 |
| HHs know about the symptoms of Covid-19 | 0.57 | 0.55 | 0.62 | 0.73 | 0.55 |
| HHs know about the quarantine period | 0.69 | 0.66 | 0.56 | 0.62 | 0.69 |
| HHs know about the testing procedure of Covid-19 | 0.80 | 0.32 | 0.26 | 0.44 | 0.48 |
| Exposure Index | 0.76 | 0.56 | 0.60 | 0.71 | 0.64 |

**Source: Field Survey, 2020**

**Table 3: Village wise sensitivity index**

| Indicators | Virjapur | Nawada | Narholi | Adooki | Bad |
| --- | --- | --- | --- | --- | --- |
| HHs belongs to below poverty line | 0.23 | 0.32 | 0.37 | 0.28 | 0.27 |
| Household head migrated in search of employment in the Covid-19 affected cities | 0.30 | 0.15 | 0.11 | 0.18 | 0.13 |
| Household head returned from Covid-19 affected cities | 0.80 | 0.90 | 0.60 | 0.50 | 0.12 |
| HHs don’t have all seasonal house | 0.23 | 0.32 | 0.27 | 0.42 | 0.43 |
| HHs don’t have access of clean water | 0.40 | 0.17 | 0.27 | 0.21 | 0.19 |
| HHs don’t have access of sanitation | 0.23 | 0.24 | 0.28 | 0.21 | 0.19 |
| Household-head has illiterate | 0.31 | 0.34 | 0.39 | 0.37 | 0.29 |
| Female-headed households | 0.30 | 0.15 | 0.19 | 0.17 | 0.16 |
| HHs don’t have electricity connection | 0.90 | 0.17 | 0.26 | 0.23 | 0.19 |
| HHs don’t have access of government health services | 0.45 | 0.90 | 0.17 | 0.14 | 0.12 |
| HHs belongs to backward social group | 0.80 | 0.67 | 0.41 | 0.49 | 0.39 |
| HHs received financial assistance from government during lockdown | 0.20 | 0.50 | 0.50 | 0.40 | 0.30 |
| HHs get food during lockdown | 0.20 | 0.12 | 0.18 | 0.16 | 0.14 |
| HHs participated in public gathering during lockdown | 0.30 | 0.30 | 0.50 | 0.40 | 0.20 |
| HHs don’t have functional bank account | 0.49 | 0.26 | 0.49 | 0.41 | 0.32 |
| Sensitivity Index | 0.41 | 0.37 | 0.33 | 0.30 | 0.23 |

**Source: Field survey, 2020**

**Table 4: Village wise adaptive capacity index**

| Indicators | Virjapur | Nawada | Narholi | Adooki | Bad |
| --- | --- | --- | --- | --- | --- |
| Household head has assured income (government employee) | 0.25 | 0.12 | 0.38 | 0.56 | 0.21 |
| HHs have agricultural land | 0.35 | 0.34 | 0.34 | 0.68 | 0.78 |
| HHs have taken health insurance | 0.25 | 0.47 | 0.45 | 0.18 | 0.12 |
| HHs have taken balance diet as a combat strategy | 0.14 | 0.34 | 0.24 | 0.39 | 0.26 |
| HHs have tested against covid-19 | 0.10 | 0.02 | 0.03 | 0.01 | 0.01 |
| Household head don’t affected to respiratory diseases | 0.21 | 0.12 | 0.36 | 0.34 | 0.88 |
| HHs have stored of all necessary food items before lockdown period | 0.24 | 0.21 | 0.28 | 0.48 | 0.43 |
| HHs have majority of young population | 0.20 | 0.56 | 0.61 | 0.59 | 0.42 |
| HHs have frequently consulted with doctors on Covid-19 | 0.20 | 0.25 | 0.45 | 0.26 | 0.59 |
| HHs have adopted self-quarantine during lockdown period | 0.27 | 0.64 | 0.58 | 0.69 | 0.80 |
| Adaptive Capacity Index | 0.22 | 0.31 | 0.37 | 0.42 | 0.45 |

**Source: Field survey, 2020**

**References**

Abid, M., Scheffran, J., Schneider, U. A., & Ashfaq, M., Farmers’ perceptions of and adaptation strategies to climate change and their determinants: The case of Punjab province, Pakistan. Earth System Dynamics, 6(2015), 225–243.

Fellmann, T., The assessment of the socio-economic impacts of climate change at household level and policy implications. In Building resilience for adaptation to climate change in the agriculture sector. Rome: Agriculture and Food Organization (2012).

Gallopin, G. C., Linkages between vulnerability, resilience, and adaptive capacity. Global Environmental Change, 16(2006), 293–303.

Hahn, M. B., Riederer, A. M., Foster, S. O., The livelihood vulnerability index: A pragmatic approach to assessing risks from climate variability and change – A case study in Mozambique, Glob. Envir. Cha. 19(2009).

IPCC, Climate change 2001: impacts, adaptation, and vulnerability: Contribution of working group II to the third assessment report of the intergovernmental panel on climate change. Cambridge University Press (2001).

Islam, M. M., Sallu, S., Hubacek, K., & Paavola, J., Vulnerability of fishery-based livelihoods to the impacts of climate variability and change: Insights from coastal Bangladesh. Regional Environmental Change, 14(2013), 281–294.

Miranda, L., Hordijk, M., & Molina, R. K. T., Water governance key approaches: An analytical framework. Literature Review, Change & Sustain, 4(2011), 1–23.

Masud, M. M., Azam, M. N., Mohiuddin, M., Banna, H., Akhtar, R., Alam, A. S. A. F., & Begum, H., Adaptation barriers and strategies towards climate change: Challenges in the agricultural section. Journal of Cleaner Production, 156 (2017), 698–706.

Nadeem, S., Elahi, I., Hadi, A., & Uddin, I., Traditional knowledge and local institutions support adaptation to water-induced hazards in Chitral, Pakistan. Kathmandu, Nepal: The International Centre for Integrated Mountain Development (ICIMOD) (2009).

Singh, S., Bridging the gap between biophysical and social vulnerability in rural India: a community livelihood vulnerability approach, A. Dev. Pol. 5(2020), 1-24.

**Structured Schedule**

**Understanding social and health vulnerability in rural India: A Case of Covid-19**

**1. General Information**

| Name of respondent |  | Village |  |
| --- | --- | --- | --- |
| State |  | Age |  |
| District |  | Social group |  |
| Block |  | Religion |  |

**2. Household Profile**

| Name of the family member | Relation to head | Age | Marital  status | Education level | occupation | Annual income |
| --- | --- | --- | --- | --- | --- | --- |
|  |  |  |  |  |  |  |
|  |  |  |  |  |  |  |
|  |  |  |  |  |  |  |
|  |  |  |  |  |  |  |
|  |  |  |  |  |  |  |

Codes: marital status-married= 1, unmarried= 2; education level- illiterate= 1, literate= 2; occupation- government employee= 1, private employee= 2.

**3. Personal attributes**

1. Do you perceive that frequencies of health-hazards has increased over past five years

A. Yes B. No

2. Do you perceive that Covi-19 has a health-hazard

A. Yes B. No

3. Do you know about the symptoms of Covid-19

A. Yes B. No

4. Do you know about the quarantine period

A. Yes B. No

5. Do you know about the testing procedure of Covid-19

A. Yes B. No

6 Do you belongs to below poverty line

A. Yes B. No

7. Does head of households out of house (migrated) in search of employment in the Covid-19 affected cities

A. Yes B. No

8. Does Household head returned from Covid-19 affected cities

A. Yes B. No

9. Do you have all seasonal house

A. Yes B. No

10. Do you have 24*7 access of clean water

A. Yes B. No

11. Do you have latrine facility within premises (sanitation)

A. Yes B. No

12. Do you have electricity connection

A. Yes B. No

13. During Covid-19 epidemic, do you have access of government health services

A. Yes B. No

14. Did you received financial assistance from government during lockdown

A. Yes B. No

15. Did you get food from government/NGOs during lockdown

A. Yes B. No

16. Did you participated in public gathering during lockdown

A. Yes B. No

17. Do you have functional bank account, so that you can avail government subsidy

A. Yes B. No

18. Do you have agricultural land

A. Yes B. No

19. Do you have health insurance

A. Yes B. No

20. Do you have taking balance diet as a combat strategy

A. Yes B. No

21. Did you have tested against covid-19

A. Yes B. No

22. Does Household head has medical history (affected to respiratory diseases)

A. Yes B. No

23. Did you have stoked of all necessary food items before lockdown period

A. Yes B. No

24. Did you have frequently consulted with doctors on Covid-19

A. Yes B. No

25. Do you self-quarantined during lockdown period

A. Yes B. No

Signature of the respondents
